# Pyogenic Sacroiliitis and Pyomyositis in a Patient with Systemic Lupus Erythematous

**DOI:** 10.1155/2014/925961

**Published:** 2014-08-03

**Authors:** Wafa Chebbi, Saida Jerbi, Wassia Kessomtini, Asma Fradi, Baha Zantour, Mohamed Habib Sfar

**Affiliations:** ^1^Department of Internal Medicine, University Hospital Taher Sfar, 5100 Mahdia, Tunisia; ^2^Department of Radiology, University Hospital Taher Sfar, 5100 Mahdia, Tunisia; ^3^Department of Physical Medicine, University Hospital Taher Sfar, 5100 Mahdia, Tunisia

## Abstract

Pyogenic sacroiliitis and pyomyositis are uncommon infectious diseases and their diagnoses are often delayed. They are typically seen in children and young adults and are rare in middle-aged people especially in those affected by rheumatic diseases. We present the first case of a Staphylococcus aureus related pyogenic sacroiliitis associated with iliacus and gluteal pyomyositis occurring in a patient with systemic lupus erythematosus. Antibiotic treatment was administered for a total of 6 weeks with a total recovery. Pyogenic sacroiliitis and pyomyositis, although remaining rare events, should be remembered as severe complications in immunosuppressed patients with inflammatory diseases. Early clinical suspicion, imaging diagnosis, and adequate therapy are decisive for the satisfactory outcome.

## 1. Introduction

Pyogenic sacroiliitis and pyomyositis are very rare infectious diseases. Diagnoses are often delayed because of their variable clinical presentations, low suspicion by the examining physician, and rare findings on radiographs. They are typically seen in children and young adults and are rare in middle-aged people especially in those affected by rheumatic diseases [1].

Systemic lupus erythematosus (SLE) is a chronic inflammatory condition associated with systemic features and multiple organ involvement. It is an immunodepression condition due to both the disease itself and its medicamentous treatment. Thus, SLE patients are at risk for developing infections [2].

We report a case of a* Staphylococcus aureus* related pyogenic sacroiliitis associated with iliacus and gluteal pyomyositis occurring in a patient with SLE.

## 2. Case Report

A 52-year-old woman was diagnosed to have SLE according to the American College of Rheumatology 1997 criteria (photosensitivity, malar rash, polyarthritis, leucolymphopenia, and positive antinuclear and anti-DNA native antibodies) and Sjögren's syndrome in April 1999. She was treated with hydroxychloroquine and corticosteroids 10 mg/day. In August 2012, she developed lower-extremity edema, and her urinalysis showed an active urine sediment and proteinuria of 1.4 g/24 h. The serum creatinine was normal (0.82 mg/dL). Both C3 and C4 titers were low. SLE disease activity index (SLEDAI) was 13. A renal biopsy showed a focal proliferative lupus nephritis, World Health Organization (WHO) class III, and International Society of Nephrology/Renal Pathology Society (ISN/RPS) class III(A), with mild activity and essentially no chronicity. Treatment with intravenous methylprednisolone (1 g/day for three days) followed by oral prednisolone with a daily dose of 60 mg and monthly pulses of intravenous cyclophosphamide for six months was started. After 8-month follow-up, her lupus nephritis was in remission on prednisone 10 mg/day, hydroxychloroquine, and mycophenolate mofetil (1.5 g/day).

**Figure 1:**
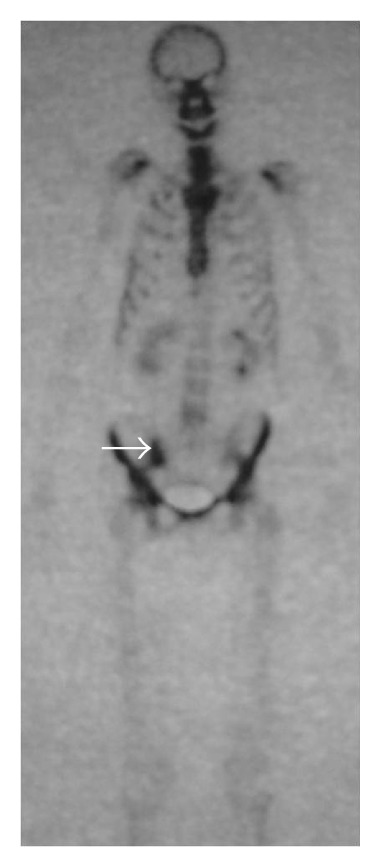
Technetium-99m MDP bone scintigraphy of the anterior pelvis shows increased uptake in the right sacroiliac joint (arrow).

**Figure 2:**
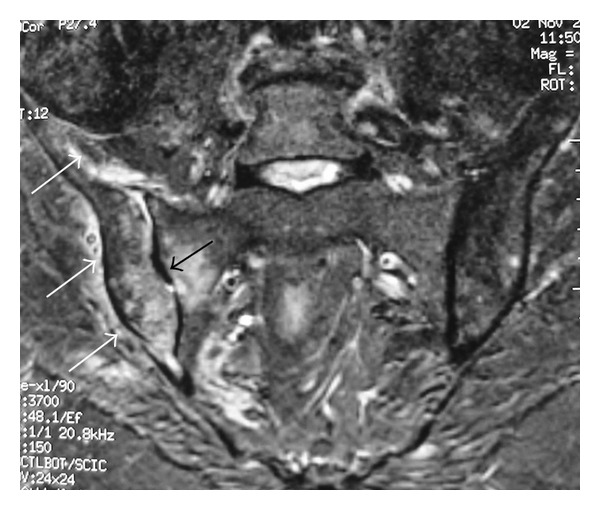
Coronal T2-weighted fat-suppressed MR image of the pelvis shows an increased signal intensity of the right sacroiliac joint (black arrow) and diffuse hyper intensity of the adjacent iliacus and gluteal muscles (white arrows).

**Figure 3:**
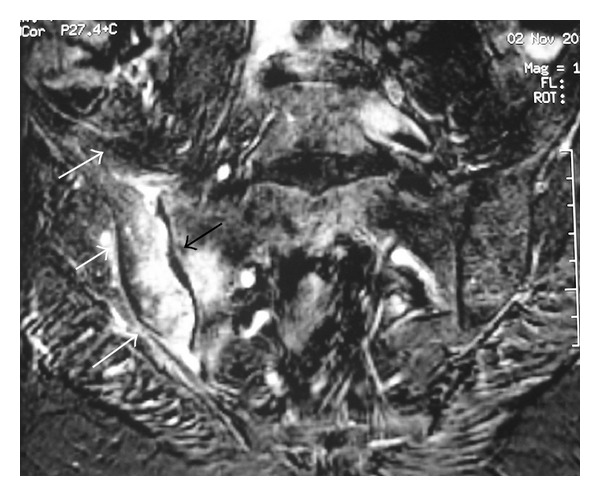
Post-contrast coronal T1-weighted MR image of the pelvis shows areas of diffuse enhancement of the right sacroiliac joint and the adjacent iliacus and gluteal muscles.

In October 2013, she was admitted with a 7-day history of acute pain, in the right hip area and buttock, and fever. The pain was progressively worsened to the point that she was unable to walk. The patient had no history of prior trauma, genitourinary problems, illicit drug use, or recent infection but she reported intramuscular injections of nonsteroidal anti-inflammatory drugs two days preceding the onset of symptoms. She was on combination of prednisolone (7.5 mg/day), hydroxychloroquine (400 mg/day), and mycophenolate mofetil (1.5 g/day). Physical examination disclosed tenderness over the right hip, and limitation of movement and pelvic compression elicited severe pain in the right sacroiliac joint. The patient had a temperature of 38.6°C, a blood pressure of 120/70 mm Hg, and a pulse rate of 100 beats per minute. There was no cutaneous lesion, adenopathy, erythema, or induration. Chest, abdominal, neurological, and gynecological examinations were normal. SLEDAI at this time was 2. Laboratory investigations showed a C-reactive protein level of 86.4 mg/L (normal range, <6 mg/L), an elevated erythrocyte sedimentation rate (108 mm/h), a white blood cell count of 8100 *μ*L, haemoglobin of 10.7 g/dL, and platelet count of 320000 ×  *μ*L. The liver and renal function tests were within normal ranges. Radiographs of the chest and right hip were unrevealing. Intravenous antibiotherapy (cefotaxime with fosfomycin) was initiated for suspected septic arthritis of the right hip or right pyogenic sacroiliitis. Technetium-99 methylene diphosphonate (Tc-99m MDP) bone scintigraphy performed two days after admission revealed increased uptake in the right sacroiliac joint (Figure 1). Magnetic resonance imaging (MRI) of the pelvis performed ten days after admission revealed an increased signal intensity of the right sacroiliac joint and diffuse hyper intensity of the adjacent iliacus and gluteal muscles on coronal T2-weighted fat-suppressed image (Figure 2). Post-contrast coronal T1-weighted MRI of the pelvis shows areas of diffuse enhancement of the right sacroiliac joint and the adjacent iliacus and gluteal muscles (Figure 3). Blood cultures disclosed* Staphylococcus aureus* sensitive to cefotaxime and fosfomycin. Tuberculin skin test and serological tests for* Salmonella* and* Brucella* were negative. A transesophageal echocardiography was normal. Treatment was continued with the same antibiotics, with disappearance of fever and improvement of pain. Rehabilitation programme was initiated so that the patient might recover her strength and mobility. The patient responded promptly to 30 days of intravenous antibiotherapy followed by an additional two weeks of oral antibiotherapy (ofloxacin). A significant improvement in her blood parameters (CRP: 2 mg/L, erythrocyte sedimentation: 18 mm/h) was obtained. Followed up six months later, the patient improved well without sequelae.

## 3. Discussion

This case deals with pyogenic sacroiliitis and iliacus and gluteal pyomyositis in a patient with SLE. The patient experienced acute pain in the right hip area and fever 2 days after intramuscular injections of nonsteroidal anti-inflammatory drugs. Infection was suspected and the patient was treated with cefotaxime and fosfomycin. The MRI confirmed the diagnosis of sacroiliitis and showed an iliacus and gluteal myositis. Additionally,* Staphylococcus aureus* was discovered in the blood culture. Antibiotic treatment was administered for a total of 6 weeks with a total recovery.

Pyogenic sacroiliitis and pyomyositis are very rare infections especially among rheumatic diseases and no case in SLE patients has been described up to now. To the best of our knowledge, our case is the first one reported in course of SLE and again it stresses the likely predisposing role played by glucocorticoids and immunosupressive treatment in a patient whose the underlying disease state may have contributed to the development of infectious complication.

Pyogenic sacroiliitis is a rare disorder, affecting between 1% and 2% of all patients with septic arthritis, which is probably due to the poor vascularisation of this joint, resulting in a low risk of infection via the haematogenous route [1, 8]. The diagnosis is difficult and often delayed owing to its clinical heterogeneity and the lack of symptom specificity [9]. The mean age of presentation is twenty years but approximately one third of the cases occur in children. The sacroiliac joint is a complex joint and an integral component of the spinal axial support system. It is a true synovial (diarthrodial) joint with a capsule and synovial fluid and, thus, subject to various forms of arthritis. Hyalin cartilage is found on the sacral side of the joint and fibrocartilage on the iliac contribution of the joint [10]. The pathophysiology of pyogenic sacroiliitis is presumed to be the hematogenous spread of bacteria from a distant source of infection to the sacroiliac joint [11]. The joint space is invaded by bacteria by either direct penetration, hematogenous, or by nearby structures such as the gut. The subchondral circulation on the iliac side of the sacroiliac joint is an end-arterial site and so may act as an entry point for inoculation of organisms with subsequent extension into the joint. Other mechanisms include direct invasion of the joint capsule or iatrogenic infection following surgical intervention or invasive procedures [12]. In adults, the most common predisposing risk factors are intravenous drugs use, pelvic trauma, infectious endocarditis, haemoglobinopathy, immunosuppressive treatment, and infections of the skin, respiratory, gastrointestinal, gynecological, and genitourinary tracts [8, 11].

Pyomyositis is a bacterial infection of the skeletal muscle. The etiology of pyomyositis is frequently classified as primary or secondary to a contiguous infection of the skin, bone, or soft tissue. Pyomyositis is most common in tropical areas, and there is a high rate of incidence in children, especially ages 2–5 [13, 14]. The pyomyositis is believed to be a complication of transient bacteremia, which is sometimes associated with a concomitant muscle tissue structure abnormality after trauma or exercise creating a locus minoris resistentiae for implantation of bacteria [13]. The most prevalent organism is* Staphylococcus aureus* with the bacteria accounting for 50–90% of cases [13, 15, 16]. Individuals with a history of diabetes, alcoholism, drug abuse, HIV infection, cancer, and systemic sclerosis are at higher risk for pyomyositis [15]. Association of pyomyositis and SLE is very rare and is limited to only a few case reports (Table 1) [3–7]. All the patients were females and made full recovery.* Staphylococcus aureus* is the most causative agent [3–6]. At the time of the event, three patients received prednisolone associated with cyclophosphamide in one case [4, 5, 7].

Pyomyositis is characterized by a three-stage clinical course beginning with a subacute stage. The second stage is formation of a muscle abscess with associated local and systemic findings. The diagnosis is most often confirmed at this stage as the involved muscle becomes increasingly swollen and tender. If not adequately treated, the third stage is reached, which includes toxicity and septic shock [13, 14, 17]. Our case presented in the second stage of iliacus pyomyositis associated with septic sacroiliitis and responded well to antibiotics. MRI is the most sensitive investigation to confirm the diagnosis. It shows increased signal intensity on T2 weighted images within the affected muscle in addition to a rim of fluid intra/perimuscular. It will also show if the infection has spread from any nearby joints [13, 17]. The hip and the muscles of the lower part of the body are most prone to pyomyositis. The muscles most commonly affected are quadriceps, gluteus, and psoas muscles [15]. Pyomyositis rarely affects the iliacus muscle and very few cases have been reported in the literature [14–16, 18–22]. Most cases occurred in children, and the diagnosis of septic arthritis of the hip was initially suspected in the majority of cases. Moreover, in two patients, both conditions, pyomyositis of the iliacus muscle and septic arthritis of the hip, presented simultaneously [17, 18]. Pyogenic sacroiliitis has been reported to be a complication of primary pyomyositis after 2 weeks of adequate treatment only in one case [15].

In our case, it is difficult to determine which occurred first, pyogenic sacroiliitis or pyomyositis. It seems more likely that pyomyositis of the iliacus and gluteal muscle first occurred, followed by infection of the sacroiliac joint. Intramuscular injections may have been the trauma and facilitated the* Staphylococcus aureus* colonisation. Therefore, we cannot exclude the possibility that pyomyositis and pyogenic sacroiliitis occurred due to hematogenous origin. The use of glucocorticoids and immunosuppressive treatment increases the possibility of infection.

In conclusion, pyogenic sacroiliitis and pyomyositis, although remaining rare events, should be remembered as severe complications in immunosuppressed patients with inflammatory diseases. Their insidious onset can delay diagnosis, limiting prognosis. MRI is the most valuable diagnostic tool and should be utilised in the early stage of the process. Early clinical suspicion, imaging diagnosis, and adequate therapy are decisive for the satisfactory outcome of such cases.

## Figures and Tables

**Table 1:** Clinical features of pyomyositis in patients with systemic lupus erythematous.

Reference	Age/sex	Country	Therapy	Muscles	Organism	Antibiotics	Outcome
Dede et al. [3]	23/F	Turkey	HCQ	Gastrocnemius	*S. aureus *	yes	Recovery
Claudepierre et al. [4]	32/F	France	Pred + Cyclo	Quadriceps	*S. aureus *	4 weeks	Recovery
Ravindran and Duke [5]	34/F	UK	Pred + HCQ	Pronator teres	*S. aureus *	4 weeks	Recovery
Souza et al. [6]	25/F	Brazil	—	Iliacus muscle	*S. aureus *	6 weeks	Recovery
Baaj et al. [7]	47/F	Morocco	Pred	Quadriceps	*E. coli *	3 weeks	Recovery
Present case	52/F	Tunisia	HCQ + Pred + MMF	Iliacus, gluteal	*S. aureus *	6 weeks	Recovery

F: female; HCQ: hydroxychloroquine; Pred: prednisolone; Cyclo: cyclophosphamide; MMF: mofetil mycophenolate; S: staphylococcus; E: *Escherichia*.
